# B Cell Depletion Eliminates FVIII Memory B Cells and Enhances AAV8-coF8 Immune Tolerance Induction When Combined With Rapamycin

**DOI:** 10.3389/fimmu.2020.01293

**Published:** 2020-06-24

**Authors:** Moanaro Biswas, Brett Palaschak, Sandeep R. P. Kumar, Jyoti Rana, David M. Markusic

**Affiliations:** ^1^Herman B Wells Center for Pediatric Research, Department of Pediatrics, Indiana University School of Medicine, Indianapolis, IN, United States; ^2^Department of Pediatrics, University of Florida, Gainesville, FL, United States

**Keywords:** hemophilia A, inhibitors, gene therapy, AAV, anti-CD20, rapamycin

## Abstract

Hemophilia A is an inherited coagulation disorder resulting in the loss of functional clotting factor VIII (FVIII). Presently, the most effective treatment is prophylactic protein replacement therapy. However, this requires frequent life-long intravenous infusions of plasma derived or recombinant clotting factors and is not a cure. A major complication is the development of inhibitory antibodies that nullify the replacement factor. Immune tolerance induction (ITI) therapy to reverse inhibitors can last from months to years, requires daily or every other day infusions of supraphysiological levels of FVIII and is effective in only up to 70% of hemophilia A patients. Preclinical and recent clinical studies have shown that gene replacement therapy with AAV vectors can effectively cure hemophilia A patients. However, it is unclear how hemophilia patients with high risk inhibitor *F8* mutations or with established inhibitors will respond to gene therapy, as these patients have been excluded from ongoing clinical trials. AAV8-*coF8* gene transfer in naïve BALB/c-*F8e16*^−/Y^ mice (BALB/c-HA) results in anti-FVIII IgG1 inhibitors following gene transfer, which can be prevented by transient immune modulation with anti-mCD20 (18B12) and oral rapamycin. We investigated if we could improve ITI in inhibitor positive mice by combining anti-mCD20 and rapamycin with AAV8-*coF8* gene therapy. Our hypothesis was that continuous expression of FVIII protein from gene transfer compared to transient FVIII from weekly protein therapy, would enhance regulatory T cell induction and promote deletion of FVIII reactive B cells, following reconstitution. Mice that received anti-CD20 had a sharp decline in inhibitors, which corresponded to FVIII memory B (B_mem_) cell deletion. Importantly, only mice receiving both anti-mCD20 and rapamycin failed to increase inhibitors following rechallenge with intravenous FVIII protein therapy. Our data show that B and T cell immune modulation complements AAV8-*coF8* gene therapy in naïve and inhibitor positive hemophilia A mice and suggest that such protocols should be considered for AAV gene therapy in high risk or inhibitor positive hemophilia patients.

## Introduction

Hemophilia, a hereditary monogenic x-linked inherited coagulation disorder, is defined by a loss in functional coagulation factor VIII (FVIII), hemophilia A, or factor IX (FIX), hemophilia B, proteins. Hemophilia A is approximately four times more common than hemophilia B with indices of 1 in 5,000 and 1 in 20,000 male births, respectively. Patients are classified as severe, moderate, or mild depending on residual coagulation factor activity and are at risk for developing spontaneous (severe) and trauma induced bleeds (moderate and mild) (1). Often bleeds occur in joints and results in hemarthrosis with significant morbidity. Bleeds into closed spaces, such as the cranium, that are not managed, result in mortality (2).

Hemophilia is presently treated on-demand or prophylactically with intravenous infusion of plasma derived or recombinant factor protein (3). More recently, extended half-life FVIII and FIX products have been made available, reducing the frequency of infusions (4). However, the development of anti-drug antibodies, termed inhibitors, remains a major complication in therapy. Inhibitor incidence is much higher in hemophilia A patients at 25–30%, whereas only 3–5% of hemophilia B patients go on to develop inhibitors (5). Often inhibitors occur in patients with severe disease, in which there is little or no expressed clotting factor. Inhibitors develop within the first year or two of starting protein therapy, often by 20 exposure days, and the relative risk of inhibitor formation is reduced with successive event free clotting factor infusions or exposure days (6).

Gene replacement therapy using adeno-associated virus (AAV) based vectors to deliver functional *F8* and *F9* genes to the liver have resulted in stable and therapeutic FVIII and FIX protein levels in adult hemophilia patients (7), with several candidates advancing into phase III clinical trials (8). Importantly, no patient receiving AAV gene therapy (despite a variability in factor protein expression levels) has developed inhibitors (7, 9, 10). However, the outcome of AAV gene therapy in young children with lower exposure days and in adults with established inhibitors is presently unknown, although the latter is scheduled to be addressed in a clinical trial (NCT03734588).

The ongoing AAV gene therapies for hemophilia are dependent on decades long preclinical studies in genetic knockout mice and naturally occurring canine hemophilia animal models (11–13). Early pre-clinical studies for hemophilia B showed that restricted expression of FIX protein to hepatocytes resulted in stable inhibitor free expression in both murine and canine hemophilia B models with a *F9* gene deletion (11). In mice, it was shown that hepatocyte restricted expression of FIX protein resulted in the induction of peripheral regulatory T cells (Treg) that suppressed the formation of inhibitory antibodies (14, 15). Later studies demonstrated that AAV liver gene therapy and these suppressive Treg could also eliminate inhibitors in murine and canine hemophilia A and B models (16–18).

While these studies suggest that gene therapy could be administered to patients with established inhibitors, it is unknown whether there is an inhibitor threshold above which gene therapy could be rendered ineffective. Combinatorial treatment with gene therapy and drugs such as rituximab could potentiate inhibitor elimination, thus preventing neutralization of the newly expressed clotting factor. Immune tolerance induction with rituximab as single-agent therapy has shown mixed responses in inhibitor patients (19, 20), with a main mechanism of action being memory B cell depletion (21). We have previously shown that combining a murine equivalent of rituximab (anti mouse CD20) with the T cell targeting drug, rapamycin can effectively reduce inhibitors in hemophilia A mice (22). We therefore applied this combination treatment regimen to gene therapy for hemophilia A in mice with established inhibitors for this study.

## Materials and Methods

### Mice

All animals used at initiation of experiments were 8 to 10-week-old male mice on the BALB/c [H-2^d^], C3H/HeJ [H-2^k^], or B6;129S [mixed H-2^b^] background. Wild type mice were purchased from Jackson Laboratories (Bar Harbor, ME). Hemophilia A mice with a deletion in exon 16 of the *F8* gene (BALB/c F8e16^−/Y^) were kindly provided by Dr. David Lillicrap (Queens, Ontario, Canada). Hemophilia B mice with a targeted deletion of murine F9 have been bred on BALB/c background for >10 generations (14). B6;129S-*F8*^*tm*1*Kaz*^ (B6;129S-HA) mice (23) were purchased from Jackson Laboratories (Bar Harbor, ME) stock number 004424 and bred in house.

Animals were housed under specific pathogen-free conditions at the University of Florida Animal Care Service facility and Indiana University laboratory animal resources center (LARC). Food and water were given *ad libitum*. Animals were treated under Institutional Animal Care and Use Committee-approved protocols.

### Viral Vectors

For FIX gene therapy, we used AAV8-ApoE/hAAT-*hF9*, which carries the hepatocyte-specific expression cassette for hFIX (24). This cassette includes an apolipoprotein E (ApoE) enhancer/hepatocyte control region, a human a1-antitrypsin promoter, hFIX cDNA, a 1.4-kb portion of intron 1 of the *F9* gene, and the bovine growth hormone poly(A) signal. For FVIII gene therapy, we used the AAV8-ApoE-hAAT-*cohBDD-F8* vector, using a codon-optimized, human B-domain deleted F8 (cohBDD-F8) gene, which has been shown to enhance the levels of transcribed FVIII protein (25). AAV serotype 8 vectors were produced as previously described (26–28).

### Reagents

Recombinant human B domain deleted (BDD) FVIII (Xyntha) was from Pfizer (New York, NY). FVIII and FIX deficient plasma was from Haematologic Technologies (Essex Junction, VT). Anti-mCD20 IgG2a subtype (clone 18B12) was purified from transfected HEK293 cells (ATUM, Newark, CA). Rapamycin was purchased from LC laboratories (Woburn, MA). Keyhole limpet hemocyanin was purchased from Sigma Aldrich (St. Louis, MO).

### Inhibitor Establishment and Gene Therapy Tolerance Regimen

For establishment of a tolerance regimen to prevent the development of FVIII inhibitors to gene therapy, naïve BALB/c F8e16^−/Y^ mice were administered 1 × 10^11^ vg AAV8-ApoE-hAAT-*cohBDD-F8* vector, using the IV route, and divided into 4 cohorts. Cohort 1 were control mice that received vector without immune suppression. Cohorts 2–4 received immune suppression along with vector. Cohort 2 received two IV doses of 250 μg anti-mCD20 (with vector and 3 weeks later). Cohort 3 received 4 mg/kg rapamycin (3x/week) by oral gavage 2 weeks after vector administration for 4 weeks. Cohort 4 received a combination of anti-mCD20 and rapamycin. Mice from cohorts 1 and 4 were further challenged with weekly IV injections for 4 weeks with 1.5 IU BDD FVIII protein (Xyntha, Pfizer) at 8 weeks following treatment. To show that mice receiving immune suppression had an intact immune response, mice were IV injected with 100 μg of keyhole limpet hemocyanin (KLH) at 14 weeks post-AAV gene transfer and bled 3 weeks later to measure anti-KLH IgG1 levels in plasma by Enzyme linked immunosorbent assay (ELISA).

For reversal studies, high titer inhibitors were initially established in naïve BALB/c F8e16^−/Y^ mice by administering weekly IV injections of 1.5 IU BDD FVIII protein for 4 weeks. This was followed by gene therapy with 1 × 10^11^vg AAV8-ApoE-hAAT-*cohBDD-F8* vector and either anti-mCD20, rapamycin, or the combination immunosuppressive regimen was administered as indicated. Follow up analysis was carried out for a further 2 months, following which, mice were re-challenged further with weekly IV injections of 1.5 IU BDD FVIII protein for 4 weeks.

### Adoptive Transfer of Treg and FVIII Antigen Challenge

Splenocyte isolation and Treg enrichment was performed as previously described (29). Briefly, spleen cells from various treatment groups were isolated and enriched following the instructions of the mouse Treg isolation kit from Miltenyi Biotec (Carlsbad, CA). In our hands, Treg enrichment with this kit typically gives a purity of 80–90% confirmed by flow cytometry staining of CD4^+^ CD25^+^ FoxP3^+^ cells. Naïve BALB/c F8e16^−−/Y^ mice received 1 × 10^6^ Treg by tail vein injection and were immunized with a subcutaneous injection of 1.5 IU of FVIII protein emulsified in Sigma Adjuvant System (Millipore Sigma S6322, St. Louis, MO). Animals were bled 3 weeks after FVIII immunization and plasma was collected to measure anti-FVIII IgG1 antibody levels using an ELISA assay.

### Analysis of Plasma Samples

Plasma samples were collected by retro-orbital eye bleed into 0.38% sodium citrate buffer. Inhibitory antibodies to FVIII or FIX were measured by Bethesda assay as described (16, 22). Measurements were carried out in a Diagnostica Stago STart Hemostasis Analyser (Parsippany, NJ, USA). ELISA-based measurements of antibodies to FVIII or FIX were carried out as described (16, 22). FVIII or FIX activity was measured by a one stage clotting assay based on a modified activated partial thromboplastin time assay (aPTT) as described (16, 22). Percent activity is an indication of the extent a plasma sample corrects the coagulation time of FVIII or FIX deficient plasma in the assay.

### Memory B Cell ELISpot

Memory B cell enzyme-linked immunospot (ELISpot) was performed as previously described (30). Briefly, spleen cells from various treatment groups were isolated and depleted of CD138^+^ antibody secreting plasma cells using anti-CD138 microbeads (Miltenyi Biotec, Carlsbad, CA). CD138^−^ spleen cells were cultured at 1.5 × 10^6^ cells/mL in RPMI 1640 (Life Technologies) supplemented with 10% fetal calf serum (Atlanta Biologicals, Norcross, GA), 2 mM l-glutamine, 10,000 U/mL penicillin, 100 mg/mL streptomycin and 55 μM β-mercaptoethanol (Sigma-Aldrich) at 37°C for 6 days. 0.1 IU/mL BDD-FVIII was added to the cells on day 0 as indicated. Newly formed antibody secreting cells (ASCs) were detected by ELISpot assays, using the CTL-ImmunoSpot system (Shaker Heights, OH).

### Flow Cytometry Studies

Peripheral blood mononuclear cells (PBMCs) were stained with the following antibodies, B220-FITC, CD4-e450, CD8-BV510, CD25-BV605, and FoxP3-e660 using the FoxP3 staining buffer kit from eBioscience per manufacturer's instructions. Data were collected on an Attune NXT flow cytometer and cell population analysis was conducted using FCS Express 7 (De Novo software, Glendale, CA).

### Statistical Analysis

All statistical analysis was carried out using Graph Pad Prism software (La Jolla, CA) using Student's 2-tailed *T*-test, one-way ANOVA with Bonferroni's multiple comparison test, or two-way ANOVA with Tukey's multiple comparisons test. A *p* < 0.05 was considered statistically significant, and indicated as follows: ^*^p < 0.05, ^**^p < 0.01, ^***^p < 0.001, and ^****^p < 0.0001.

## Results

### Immune Response to Clotting Factor Protein or Gene Therapy Is Mouse Strain Dependent

**Figure 1:**
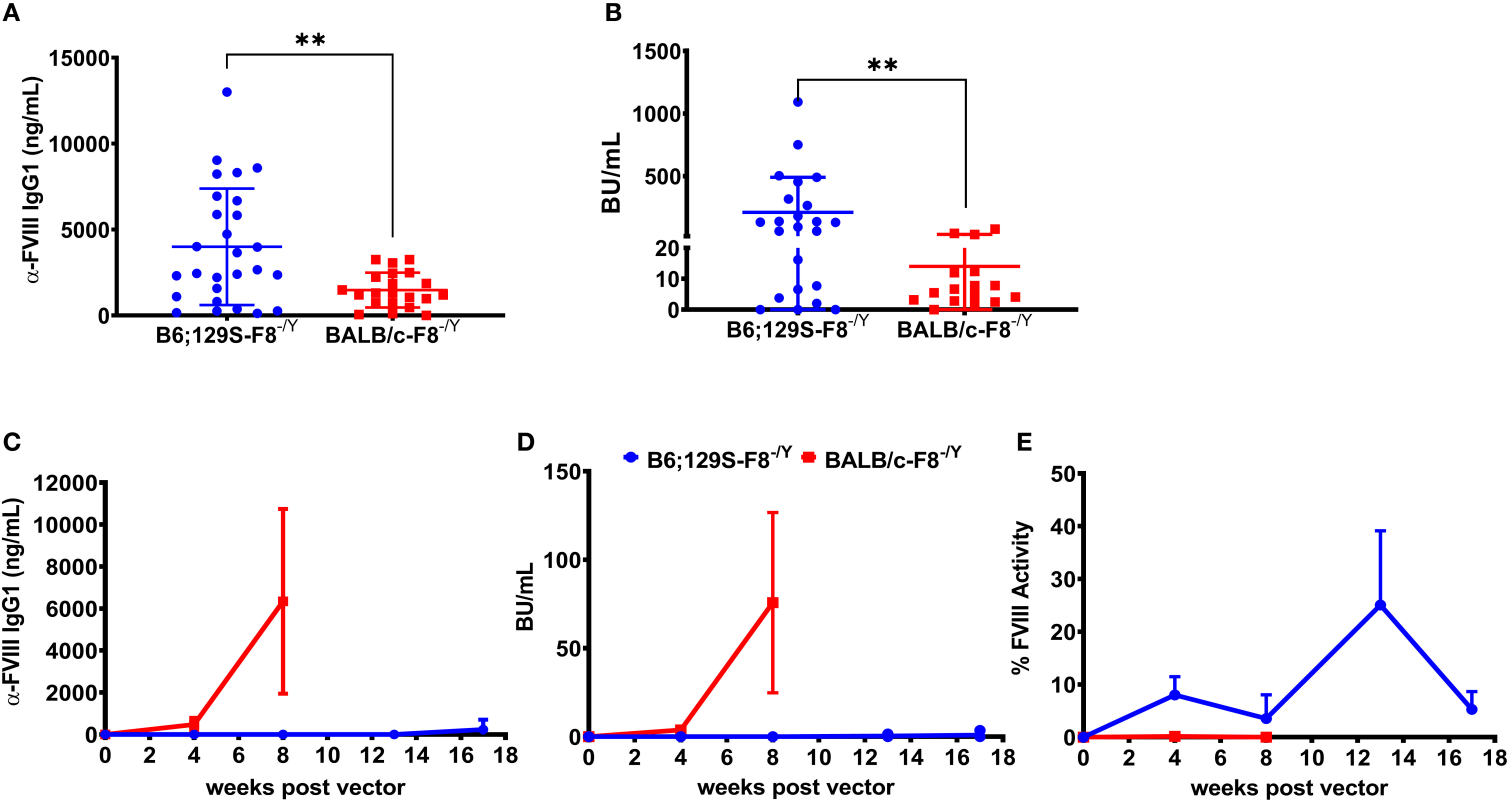
Differential immune responses to BDD-FVIII protein in hemophilia A mice on different genetic backgrounds when delivered as recombinant protein or expressed in hepatocytes. Hemophilia A mice on B6;129 (*n* = 27) or BALB/c (*n* = 21) backgrounds received 4 weekly IV injections of 1 IU BDD-FVIII and plasma was collected at week 5 to measure anti-FVIII IgG1 levels **(A)** and Bethesda inhibitor titers **(B)**. In some animals we were unable to measure Bethesda inhibitors due to technical issues. Hemophilia A mice on 129;B6 or BALB/c backgrounds (*n* = 4 per strain) were IV injected with 1 × 10^11^ vg AAV8-ApoE-hAAT-*coF8* vector and followed over time for anti-FVIII IgG1 levels **(C)**, Bethesda inhibitor titers **(D)**, and one-stage aPTT activity assay **(E)**. Mice were rechallenged with weekly IV injections of 1 IU BDD-FVIII protein for 4 weeks **(C–E)**. Statistical analysis of **(A,B)** was performed using 2-tailed Mann-Whitney test and **(C–E)** with multiple row *T*-test. A *p* < 0.05 was considered statistically significant, and indicated as follows: *p < 0.05, **p < 0.01, ***p < 0.001, and ****p < 0.0001.

Historically we have observed that the hemophilia B mice on a BALB/c background are less responsive to FIX protein therapy compared to mice on the C3H/HeJ background with an identical *F9* gene deletion (31). A previous study comparing immune responses to recombinant full length FVIII protein therapy in BALB/c and C57BL/6 hemophilia A mice reported that mice on the C57BL/6 background developed a higher antibody and inhibitor titer (32). However, more recombinant FVIII protein products are B-domain deleted FVIII (BDD-FVIII) and clinical gene therapy vectors also express BDD-FVIII protein. Thus, it is important to understand potential genetic risk factors for inhibitor formation against recombinant and vector derived BDD-FVIII. Groups of hemophilia A mice on BALB/c (BALB/c-HA) or B6;129S (B6;129S-HA) background received 4 weekly IV injections of 1 IU BDD-FVIII protein. Blood was collected on week 5 and plasma levels of anti-FVIII IgG1 and Bethesda titers were determined. We observed significantly lower levels of both anti-FVIII IgG1 (Figure 1A) and Bethesda inhibitor (Figure 1B) titers in BALB/c-HA mice. B6;129S-HA mice (*n* = 23) had a mean anti-FVIII IgG1 level of 3,996 ng/mL and inhibitor titer of 210 BU/mL compared to BALB/c-HA mice (*n* = 16) anti-FVIII IgG1 of 1,479 ng/mL and Bethesda titer of 14 BU/mL.

Based on the stronger response of B6;129S-HA mice to recombinant BDD-FVIII protein, we hypothesized that these mice would have a higher risk of developing inhibitors following gene transfer of an AAV8-ApoE-hAAT-*cohBDD-F8* (AAV8-*coF8*) vector. To test this, we injected both hemophilia A mouse strains with 1 × 10^11^ vg of the AAV8-*coF8* vector and followed mice over time. Plasma was tested at different time points post-vector injection and was analyzed for FVIII activity, Bethesda inhibitors, and anti-FVIII IgG1. Surprisingly, BALB/c-HA mice developed both anti-FVIII IgG1 antibodies (Figure 1C) and Bethesda inhibitor titers (Figure 1D) 8 weeks post-vector administration. In contrast, B6;129S-HA mice did not develop anti-FVIII IgG1 or Bethesda inhibitors (Figures 1C,D) and displayed moderate FVIII activity (Figure 1E) even after 4 weekly challenges with recombinant BDD-FVIII protein.

### Transient Immune Modulation With Anti-CD20 and Rapamycin Combination Therapy Prevents Inhibitors to FVIII Gene Therapy in BALB/HA Mice

**Figure 2:**
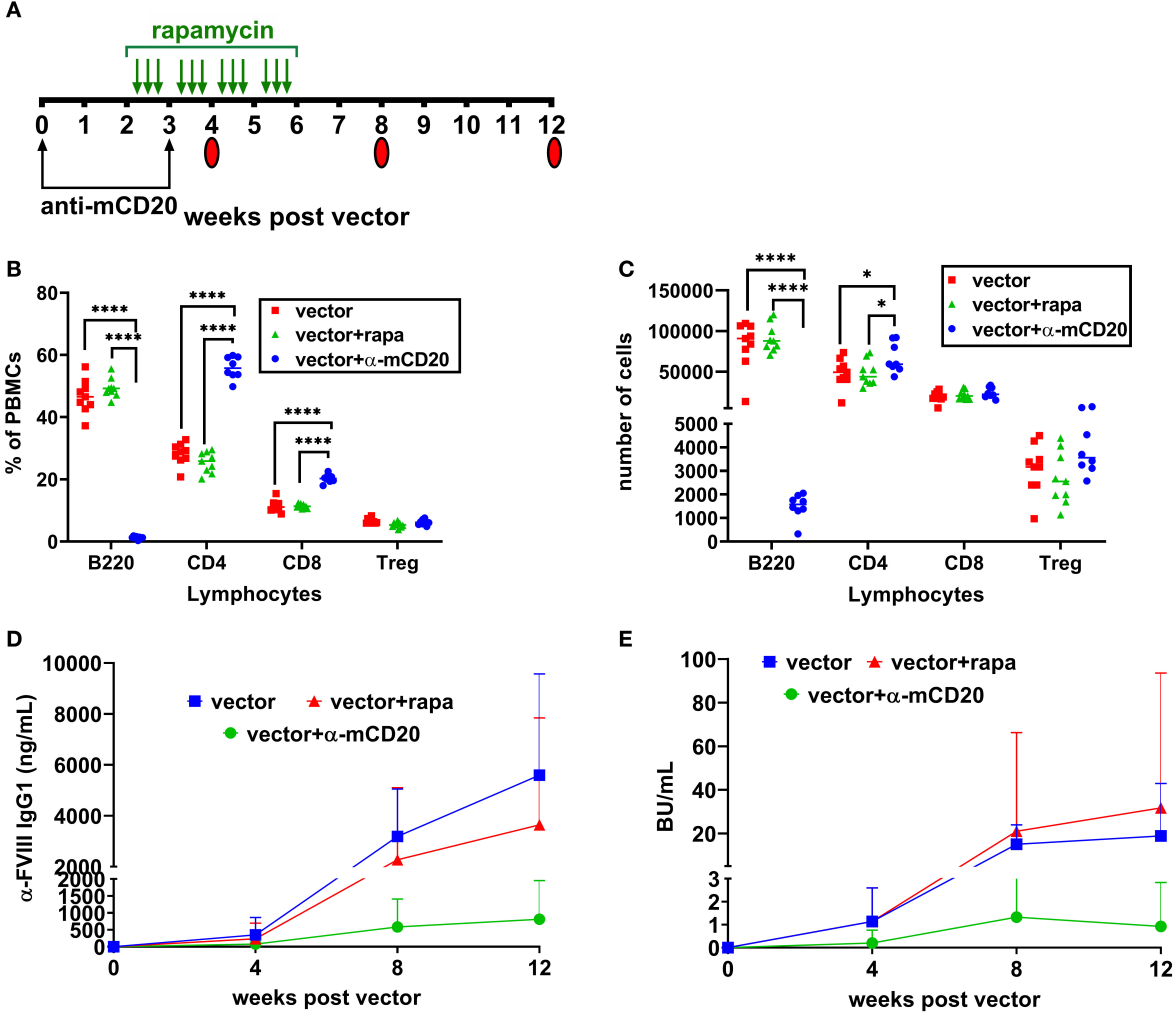
Prophylactic immune suppression with anti-mCD20 or rapamycin is only partially effective at preventing inhibitors. BALB/c-HA mice were divided into three groups (*n* = 8 per group), control (red), rapamycin (green), and anti-mCD20 (blue). All groups were IV injected with 1 × 10^11^ vg of the AAV8-*coF8* vector. Treatment and blood collection time points are indicated in the timeline **(A)**. Flow cytometry staining of PBMCs was performed at week 4 to stain for B cells (B220^+^), CD4 T cells (CD4^+^), CD8 T cells (CD8^+^), and Treg (CD4^+^ CD25^+^ FoxP3^+^), and data is reported as frequency of PBMCs **(B)** and total cell counts **(C)**. Anti FVIII IgG1 levels **(D)** and Bethesda inhibitor titers **(E)** over time. A *p* < 0.05 was considered statistically significant, and indicated as follows: *p < 0.05, **p < 0.01, ***p < 0.001, and ****p < 0.0001.

Based on these results (Figures 1C,D), we used BALB/c-HA mice that naturally develop inhibitors following AAV8-*coF8* gene therapy to determine if our immune modulatory protocol could prevent inhibitors and promote FVIII tolerance. Our group has previously developed several transient immune modulatory protocols to prevent and reverse inhibitors in the context of FVIII protein therapy in BALB/c-HA mice (22, 25, 33, 34). Thus, we hypothesized that transient immune modulation may be effective at preventing inhibitors following AAV8-*coF8* liver gene transfer. Based on our previous published studies with immune suppression in the context of protein therapy (22), we elected to initially test a B cell depleting antibody, anti-mCD20 (250 μg) on weeks 0 and 3 or oral rapamycin (4 mg/kg) 3 times per week from weeks 2 through 5 post-vector administration [Figure 2A; (22, 33)]. PBMCs were collected at week 4 post-vector administration and stained with antibodies to detect B cells (B220), CD4 and CD8 T cells, and Tregs (CD4^+^CD25^+^FoxP3^+^). As expected, we observed a significant decrease in the frequency and number of B220 positive cells in mice receiving the B cell depleting anti-mCD20 antibody compared to vector and vector and rapamycin treated mice (Figures 2B,C). Mice receiving the anti-mCD20 antibody had a statistically significant elevated frequency of CD4 and CD8 T cells but only had a modest elevation in total CD4 T cells. There was no significant difference in the frequency and total number of Tregs between the different groups (Figures 2B,C). By week 8 post-vector, the majority of mice treated with vector alone had developed FVIII inhibitors as measured by anti-FVIII IgG1 and Bethesda assay (Figures 2D,E). In contrast, about half of the mice treated with AAV and rapamycin developed inhibitors. However, one animal in this group had the highest titer inhibitor in the whole cohort, suggesting that rapamycin alone is not effective at preventing inhibitors (Figures 2D,E). AAV combined with anti-mCD20 was the most effective combination with only two animals developing inhibitors at week 8 post-vector (Figures 2D,E). At week 12 anti-FVIII IgG1 levels were starting to emerge in the AAV combined with anti-mCD20 mice. However, only two animals in the group had a detectable Bethesda inhibitor (Figures 2D,E).

**Figure 3:**
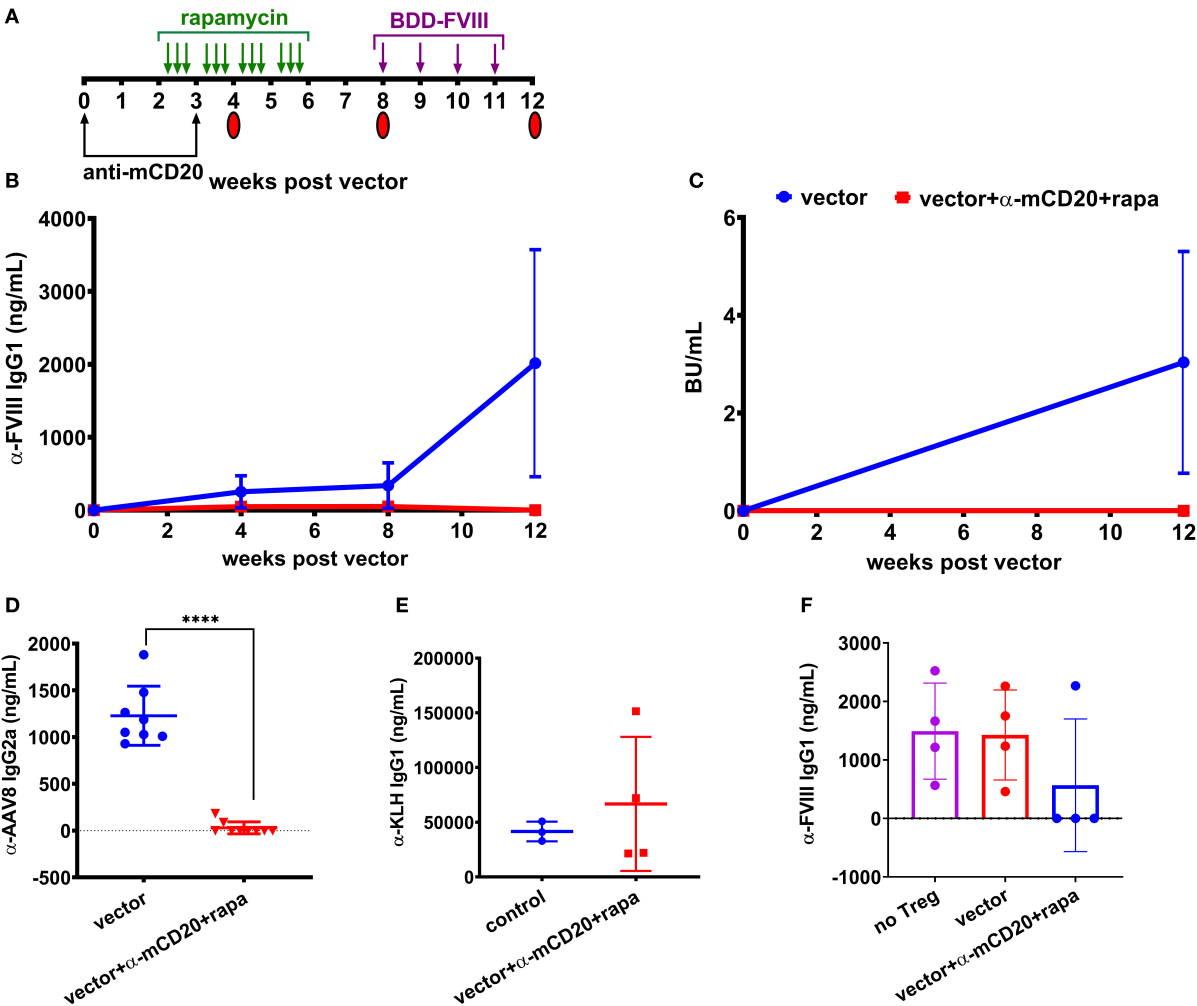
Prophylactic immune suppression with anti-mCD20 and rapamycin prevents inhibitors following AAV8-*coF8* gene delivery in BALB/c-HA mice. BALB/c-HA mice were divided into two groups (*n* = 8 per group), control (blue) and anti-mCD20 plus rapamycin treated (red). **(A)** Both groups were IV injected with 1 × 10^11^ vg AAV8-*coF8* vector as indicated in the experimental timeline. Mice receiving immune suppression received anti-mCD20 along with vector and 3 weeks later. Oral gavage of rapamycin was started 2 weeks after vector, three times per week for 4 weeks. Mice received weekly IV injections of 1.5 IU FVIII starting at week 8 for 4 weeks. Mice were followed over time for anti-FVIII IgG1 levels **(B)**, and Bethesda inhibitor titers **(C)**. Plasma from mice at 8 weeks post-vector was used to measure anti-AAV8 IgG2a antibody levels by ELISA **(D)**. The surviving mice AAV only (*n* = 3) and AAV with immunosuppression (*n* = 4) were challenged with KLH to show that the transient immunosuppression did not compromise adaptive immunity and anti-KLH IgG1 levels were measured by ELISA **(E)**. Treg were isolated from four mice in each treatment group and 1 × 10^6^ Tregs were adoptively transferred into naïve BALB/c-HA mice and the next day mice (Treg transfer and naïve controls) were challenged with subcutaneous delivery of 1.5 IU FVIII protein in adjuvant and bled 2 weeks later to measure anti-FVII IgG1 antibody levels by ELISA **(F)**. Statistical analysis of **(D)** was performed using unpaired *T*-test with ****p < 0.0001.

To improve the effectiveness of inhibitor suppression, we designed a study to combine both anti-mCD20 and rapamycin along with vector administration. Mice received weekly challenges of 1.5 IU BDD-FVIII for 4 weeks starting at 8 weeks post-vector (Figure 3A) and at the end of the challenge, some animals were followed longer while others were used as donors for regulatory T (Treg) cell adoptive transfer studies into naïve BALB/c-HA mice. During the course of treatment, blood was collected and plasma anti-FVIII IgG1 levels and Bethesda titers were measured. Control mice receiving only vector developed inhibitors whereas mice receiving immune modulatory therapy with vector failed to develop inhibitors even after immunological challenge with recombinant FVIII protein (Figures 3B,C). Additionally, 7 out of 9 mice receiving the immune modulation failed to mount an IgG2a antibody response against the AAV8 capsid (29.97 ± 64 ng/ml) whereas all control mice treated with vector alone formed anti-AAV8 IgG2a antibodies (1,229 ± 316 ng/mL, Figure 3D).

To test if immunological tolerance was established, we isolated Treg from the spleens of a subset of the BALB/c-HA mice receiving either vector alone or vector with immune modulation and adoptively transferred 1 × 10^6^ Treg into naïve BALB/c-HA mice, followed by subcutaneous challenge with 1.5 IU FVIII in adjuvant. Naïve BALB/c-HA mice were also challenged with FVIII in adjuvant to provide a baseline for the maximal antibody response. Three of four mice receiving Treg from vector/rapamycin/anti-mCD20 treated donors failed to generate antibodies against FVIII protein whereas mice receiving Treg from vector only donors were indistinguishable from control adjuvant challenged mice (Figure 3F). This demonstrates that transient immune suppression coupled with AAV liver gene delivery of FVIII protein is capable of augmenting antigen specific Treg as previously observed in the context of FVIII protein replacement therapy (29).

Finally, in order to confirm that immune suppression by vector/rapamycin/anti-mCD20 treatment was transient, we administered the strong immune stimulatory T help dependent antigen keyhole limpet hemocyanin (KLH) in mice, 2 months after immune suppressive treatment was completed. Both controls and mice that had previously received immune suppressive treatment developed robust IgG1 antibody responses to KLH (41,571 vs. 66,745 ng/mL, respectively), indicating that B and T cell compartments had completely recovered in these animals (Figure 3E).

Our prior work has shown that AAV-*F9* liver gene transfer alone is sufficient for elimination of anti-FIX IgE and IgG1 as well as functional inhibitors in C3H/HeJ-HB mice (16). Here we show that AAV8-*F9* liver gene transfer is also effective at eliminating inhibitors in BALB/c-HB mice, with the same murine *F9* deletion (Figure S1). However, in this background, inhibitor induction required subcutaneous injection of FIX protein in adjuvant as the majority of mice failed to develop significant anti-FIX IgG1 levels following recombinant FIX protein challenge with 10 IU FIX protein [Figure S1A; (31)]. A vector dose of 1 × 10^10^ vg failed to reverse inhibitors in contrast to what we had reported in C3H/HeJ-HB mice, although this result is not unexpected as a previous study demonstrated that successful inhibitor reversal in adjuvant-FIX challenged mice with a lentiviral vector required higher FIX expression levels (12). However, a higher vector dose of 1 × 10^11^ vg led to the rapid elimination of inhibitors (Figures S1B,C) and therapeutic levels of hFIX protein (Figure S1D). Over time, FIX levels in inhibitor positive BALB/c-HB mice converged with naïve BALB/c-HB treated with the same vector dose. Thus, these data demonstrate that mice on a BALB/c background are responsive to AAV liver mediated tolerance induction despite a previous report which claimed impaired tolerance in this background (35).

### Anti-CD20 and Rapamycin Therapy Tolerizes FVIII Gene Therapy in BALB/c HA Mice With Established Inhibitors

**Figure 4:**
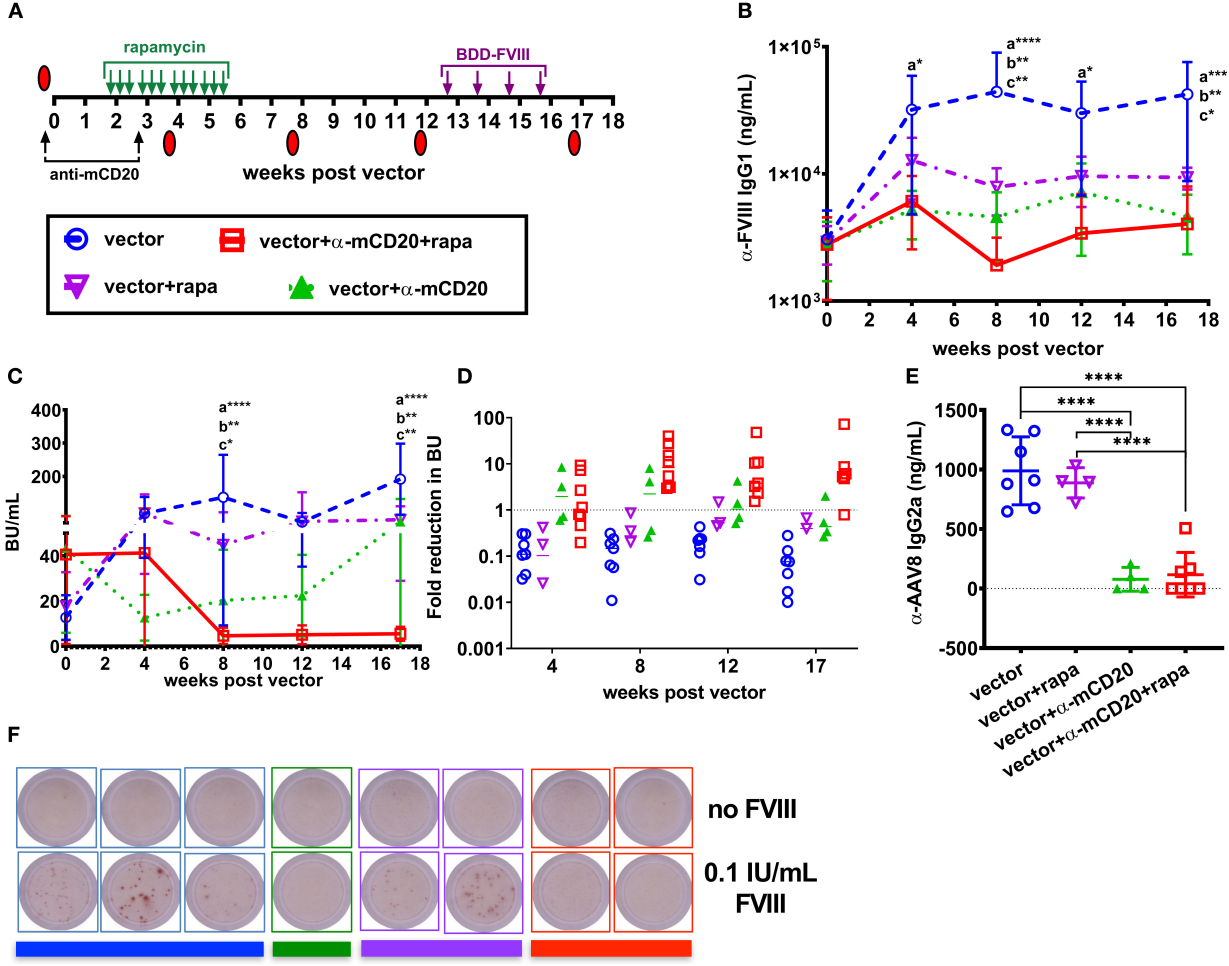
AAV8-*coF8* liver gene transfer enhances anti-mCD20 and rapamycin ITI. BALB/c-HA mice were IV challenged weekly for 4 weeks with 1.5 IU BDD-FVIII protein and bled at different time points to determine starting Bethesda inhibitor titers. Different groups were established that received vector only (1 × 10^11^ vg AAV8-ApoE-hAAT-*coF8, n* = 7 blue circle), vector and rapamycin (*n* = 5 inverted purple triangle), vector and anti-mCD20 (*n* = 5 green triangle), and vector with anti-mCD20 and rapamycin (*n* = 8 red square). Vector and anti-mCD20 was administered at week 0 and a second anti-mCD20 at week 3, denoted with black arrows. Oral gavage with rapamycin was started 2 weeks after vector, three times per week for 4 weeks (green arrows). Summary of experimental timeline **(A)**. Mice were re-challenged with weekly IV injections of 1.5 IU FVIII starting at week 13 for 4 weeks. All mice received 1 × 10^11^ vg of the AAV8-*coF8* vector by tail vein injection. Plasma was used to measure anti-FVIII IgG1 levels **(B)** and Bethesda inhibitor titers **(C)**. The fold reduction in Bethesda inhibitor titers in relation to the starting titer for each mouse and group were calculated and reported in **(D)**. Values >1 represent a reduction in Bethesda inhibitor titers, 1 no change, and <1 an increase in Bethesda inhibitor titers. Plasma from 8 weeks post-vector was used to measure anti-AAV8 IgG2a antibody levels by ELISA **(E)**. Representative wells of a FVIII memory B cell ELISpot assay in which CD138 depleted splenocytes were cultured for 6 days in the absence or presence of 0.1 IU FVIII/mL **(F)**. Colored bars beneath the wells correspond to the donor group. Statistical analysis of **(B,C)** was conducted using two-way ANOVA with Tukey's multiple comparisons test. Groups with significant differences are annotated a: vector vs. vector-anti-mCD20-rapamycin, b: vector vs. vector-anti-mCD20, and c: vector vs. vector-rapamycin with *p < 0.05, **p < 0.01, ***p < 0.001, and ****p < 0.0001. Statistical analysis of **(E)** was conducted by one-way ANOVA with Tukey's multiple comparisons test.

After establishing that anti-CD20 and rapamycin treatment complements AAV8-*coF8* liver gene transfer, we next asked if this combined therapy would be effective for ITI. To induce inhibitors, BALB/c-HA mice received 4 weekly IV injections of 1.5 IU BDD-FVIII protein and animals were bled and tested for anti-FVIII IgG1 and Bethesda titers. Mice were then divided into four groups: (1) vector alone, (2) vector plus rapamycin, (3) vector plus anti-mCD20, and (4) vector plus rapamycin and anti-mCD20, in which each group contained a similar range of inhibitor titers (Figure 4A). To determine if tolerance was established, BALB/c-HA mice received an additional 4 weekly injections of 1.5 IU BDD-FVIII protein at week 13 post-vector and were bled the following week. Mice in the vector only and vector and rapamycin treatment groups had an increase in both anti-FVIII IgG1 and Bethesda inhibitor titers (Figures 4B,C), whereas mice in the vector/anti-mCD20 and vector/rapamycin/anti-mCD20 groups had an initial decrease in both anti-FVIII IgG1 (Figure 4B) and Bethesda inhibitor titers (Figure 4C). We observed a significant decrease in anti-FVIII IgG1 levels in animals receiving immune suppression compared to only vector at weeks 4 and 17 and at all time points in mice receiving anti-mCD20 and rapamycin. A significant decrease in Bethesda inhibitor titers was observed at weeks 8 and 17 in all animals receiving immune suppression. However, only mice receiving vector/rapamycin/anti-mCD20 maintained their reduced anti-FVIII IgG1 and Bethesda inhibitor titers following BDD-FVIII protein challenge (Figures 4B,C). Overall, the average fold reduction in Bethesda titers in response to the treatment regimen was consistently higher for the vector/rapamycin/anti-mCD20 group (3.8 to 8.2-fold reduction, weeks 8–17), as compared to the vector/anti-mCD20 group (0.43 to 2.2-fold reduction, weeks 8–17, Figure 4D). Importantly, inhibitors failed to recover in the triple treated group when rechallenged with intravenous FVIII protein therapy. However, despite the reduction in inhibitor titers in the vector/rapamycin/anti-mCD20 group, we were unable to detect any functional correction in hemostasis in any of the treated animals using a two-stage chromogenic assay (data not shown).

Importantly, both the vector/anti-mCD20 and vector/rapamycin/anti-mCD20 treatment regimens reduced the formation of IgG2a antibodies to the AAV8 capsid (103 and 116 ng/mL, respectively). Mice treated with vector only or with vector/rapamycin developed anti-AAV8 IgG2a antibodies (988 and 888 ng/mL, respectively) that would theoretically preclude successful re-administration of the vector (Figure 4E).

Since only animals treated with the B cell depleting antibody, anti-mCD20 (alone or in combination with rapamycin), showed a reduction in inhibitors, we hypothesized that this was likely due to the elimination of anti-FVIII IgG1^+^ memory B cells. Our previous studies in BALB/c and BALB/c-HA mice demonstrated that a substantial fraction of the B cell compartment is eliminated following anti-mCD20 treatment (22). During the course of B cell maturation, a subset of cells become memory B cells capable of rapidly responding to secondary exposure to antigen (36). Importantly, it has been shown that FVIII specific IgG1^+^ memory B cells require the presence of activated T cells and antigen in order to differentiate into antibody producing plasma cells (37). To test this hypothesis, we isolated splenocytes from the surviving BALB/c-HA mice of each group to look at the frequency of FVIII specific memory B cells. Plasmablasts and plasma cells were depleted using anti-CD138 magnetic beads and the remaining splenocytes were cultured for 6 days with or without 0.1 IU/mL BDD-FVIII protein (16, 30, 37). Following re-stimulation, the cells were plated for a FVIII specific B cell ELISpot assay. All mice treated with anti-mCD20 were negative for FVIII memory B cells whereas mice from the other two groups that received vector alone or vector and rapamycin developed FVIII ASCs only upon antigen re-stimulation (Figure 4F). To verify these results we conducted an additional study to expand the group of vector/anti-mCD20 and vector/rapamycin/anti-mCD20 animals and again observed an absence of FVIII memory B cells, whereas control mice immunized with intravenous delivery 1.5 IU recombinant FVIII protein had a substantial population of FVIII specific memory B cells (Figure S2). Overall, our results suggest that elimination of memory B cells is critical for successful ITI with AAV8-*coF8* liver gene therapy, and that this process is enhanced by T cell suppression with rapamycin.

## Discussion

Despite the successes of recent clinical trials evaluating liver directed AAV gene therapy for hemophilia A and hemophilia B, all trial participants to date have been selected based on gene mutation and history with an absence of inhibitors. However, a newly proposed clinical study (NCT03734588) may soon be underway to address the risk of AAV gene therapy in hemophilia A patients with low titer inhibitors. Pre-clinical data in mouse, dogs, and non-human primates (NHP) support immune tolerance induction with AAV-*F9* hepatocyte gene transfer, as this restores expression at its natural site of synthesis (11, 16). However, inhibitors in hemophilia B patients undergoing FIX protein therapy is rare with an incidence of 3–5% (5) and does not likely represent a major complication in hemophilia B gene therapy.

In contrast, the incidence of inhibitors in hemophilia A patients is 25–30% meaning that a larger fraction of patients may be ineligible for gene therapy. This premise is supported by the increased incidence of inhibitor formation in pre-clinical mouse and NHP studies following AAV-*F8* gene transfer and data from the present study (11, 38). Despite the fact that hepatocytes can express functional FVIII protein, FVIII is naturally synthesized in endothelial cells, such as liver sinusoidal endothelial cells therefore expression and secretion in hepatocytes may be less than optimal (27, 39). Interestingly, pre-clinical data in hemophilia A mice show effective ITI with *in vivo* delivery of a lentiviral vector with FVIII expression restricted to LSECs (40, 41). It is therefore important to develop protocols to improve outcomes in high risk and inhibitor positive patients.

Others have reported that AAV-*F8* gene transfer in mice and non-human primates (NHP) often leads to anti-FVIII antibody formation, attributing this to expression of a human protein in a different species (42, 43). However, expression of other human proteins, such as human FIX protein, from AAV liver gene transfer in mice rarely results in inhibitor formation (14). Thus, the risk of provoking an immune response against a liver expressed protein in the context of gene therapy is likely more dependent on the relative immunogenicity of the transgene (44). This discrepancy in the tolerogenicity of *AAV-F8* and *AAV-F9* liver gene transfer, especially in the context of pre-existing inhibitors has also been documented by others (38). Data from two clinical studies in adult hemophilia A patients show that AAV-*F8* gene transfer is safe and effective and thus far no patients have developed anti-FVIII IgG antibodies and inhibitors. However, exclusion criteria for these trials eliminates patients with mutations associated with inhibitor and excludes any patient that had a history of inhibitor. Thus, it remains unknown how hemophilia A patients with limited FVIII exposure days or with inhibitors will respond to AAV8-*coF8* gene therapy.

A surprising finding in our study was that BALB/c-HA and not 129/B6-HA mice treated with an AAV8-*coF8* vector spontaneously developed anti-FVIII IgG1 inhibitors, despite BALB/c-HA mice having a weaker immune response to recombinant human BDD-FVIII protein therapy. One potential explanation is the suggestion that mice on a BALB/c background have impaired hepatic tolerance following AAV liver gene transfer (35). However, we have demonstrated that AAV-*F9* ITI therapy is highly effective at eliminating inhibitors in BALB/c-HB mice and can restore hemostasis (16). Gaining an understanding of why these mice have a differential immune response toward endogenously expressed BDD-FVIII from AAV transduced hepatocytes and recombinant BDD-FVIII protein infusion may therefore help to influence future gene therapy trial design. An alternative explanation is that the levels of BDD-FVIII protein provided by AAV gene transfer to hepatocytes in this study may not be sufficient for tolerance induction. Indeed, for FIX gene therapy, inhibitor positive BALB/c-HB mice given 1 × 10^9^, and 1 × 10^10^ vg AAV8-*F9* was insufficient for completely eliminating inhibitors. Vector dose and antigen levels have been indirectly and directly tied to an increase in antigen specific Treg (16, 45), with a small reduction in vector dose shifting the balance from tolerance to immunity (46). Recent progress with engineered and hybrid FVIII proteins may help improve BDD-FVIII transgene levels (47). However, it is presently unclear how these FVIII variants will be viewed by regulatory agencies and the immune system (48).

T helper cell dependent antibody formation, which is responsible for the majority of inhibitors in hemophilia patients, requires the activation of FVIII specific effector T cells by professional antigen presenting cells (APC) presenting small peptide epitopes of FVIII protein. In the presence of antigen, activated effector T cells, including T follicular helper cells (T_FH_) provide critical co-stimulatory signals to promote the maturation of immature B cells in B cell follicles into high affinity class switched antibody producing plasma cells. During this maturation process, a large fraction of these cells become long-lived antibody producing plasma cells, which exit the follicles and migrate to the bone marrow. However, a subset of the high affinity class switched B cells become memory B cells and persist, providing a pool of primed B cells that can rapidly differentiate into plasma cells with T cell help and antigen exposure.

Immune suppression protocols combining rapamycin and anti-CD20 have been used in the clinic to treat refractory anti-drug antibodies in hemophilia and the lysosomal storage disorder, Pompe disease (49–53). The immune modulatory protocol that we employed in this study is designed to be transient to avoid compromising long-term systemic immunity. We demonstrated that the most effective immune modulatory protocol for AAV ITI was the inclusion of both anti-mCD20 and rapamycin. Similar to our previous study evaluating anti-mCD20 and rapamycin in the context of FVIII protein therapy (22), we observed an initial decrease in anti-FVIII IgG1 levels and Bethesda inhibitor titers in both groups that were treated with anti-mCD20. One potential explanation for these results is that anti-FVIII IgG1 in our mouse model is likely produced by both plasmablasts and plasma cells. Thus, we can speculate that this initial decrease is due to elimination of plasmablasts which express CD20 and are thus sensitive to depletion with anti-mCD20. Although elimination of memory B cells played an important role in the initial reduction in inhibitor titers, lasting reduction was only achieved in mice that also received rapamycin, which has been shown to eliminate antigen specific CD4^+^ T effector cells while expanding antigen specific Treg (33). Rapamycin at high doses can also inhibit B cell proliferation and differentiation, class switching and germinal center responses (54, 55).

We hypothesized that sustained expression of FVIII protein from AAV8-*F8* transduced hepatocytes would enhance the effectiveness of ITI when combined with rapamycin and anti-mCD20. In our prophylactic immune tolerance study (Figure 3) we demonstrated that immune suppression and AAV8-*F8* vector synergized to prevent inhibitors compared to controls that received vector alone. When we adoptively transferred Tregs from different groups of donor mice into naïve BALB/c-HA, only Tregs from mice receiving immune suppression and vector were able to suppress antibody formation following re-challenge with FVIII protein in adjuvant, thus demonstrating that vector derived FVIII protein was capable of expanding FVIII specific Treg in the presence of rapamycin. We asked if vector derived FVIII protein could help improve the effectiveness of our previously published ITI protocol with rapamycin and anti-mCD20 (22). The main finding in our previous study was that immune suppression showed the best outcomes for reducing inhibitor titers in mice with starting Bethesda inhibitor titers of ~10 BU/mL. By including gene therapy to the ITI protocol we are now able to achieve significant reduction of inhibitors in mice with an average starting titer of 43 BU/mL, supporting our hypothesis that combining gene and immune suppressive therapy is an effective ITI therapy. One limitation of the study was that we were not able to determine if the improved tolerance from immune suppression with anti-mCD20 and rapamycin was transient as we challenged mice with recombinant FVIII protein 7 weeks after the last rapamycin dosing. Thus, it is possible that hemophilia mice might not remain refractory to FVIII protein challenge when there is a longer interval between immune suppression and FVIII protein challenge.

Immune modulatory therapy targeting both the B and T cell compartments displayed the best synergy with AAV-*F8* ITI in BALB/c-HA mice with established inhibitors. However, even with adjunct immunotherapy we were unable to completely eliminate inhibitors and restore hemostasis in contrast to a previous study reporting inhibitor eradication following AAV8-*cF8* gene transfer in hemophilia A dogs with inhibitor (17). The timing for elimination of inhibitors in vector treated dogs was dependent on their peak inhibitors titers and was between 4 and 5 weeks in animals <10 BU/mL and up to 18 months in a dog with a peak titer of 216 BU/mL. It is challenging for a similar long-term follow-up in mice due to their shortened lifespans. Our study went out to 17 weeks post-vector treatment and after this time point, we began to lose a substantial number of animals due to health complications, possibly due to aging or aggression related injury. Thus, we cannot rule out that inhibitor titers would have resolved with longer follow-up. However, there are several differences between our study and the one conducted in hemophilia A dogs. We used a codon optimized human *F8* cDNA compared to a dual vector expressing canine *F8* heavy and light chain. Canine FVIII has been shown to be both better expressed, have a higher specific activity, and increased stability as compared to human FVIII (56). Another consideration for the effectiveness of AAV ITI in hemophilia A is the relative proportion of long-lived plasma cells, which are CD20 negative, and thus are refractory to anti-mCD20 depletion. Even though our treatment was able to deplete memory B cells, long lived plasma cells are known to maintain serum antibody levels in the absence of memory B cells (57). One approach to address this is the inclusion of agents such as AMD3100 (plerixafor) and G-CSF to mobilize plasma cells from their survival niche in the bone marrow (58). This combined therapy could potentially increase the success of ITI and allow the inclusion of inhibitor patients for FVIII gene therapy. These drugs are commonly used in the clinic for cancer, autoimmune disease treatment, and to prevent graft rejection, and therefore are safe and validated. Additionally, the dose, route of delivery (oral intake vs. IV injection) and frequency of rapamycin administration could be adjusted to improve tolerance induction as we have previously found that daily oral rapamycin was more effective at suppressing inhibitors in the context of FVIII protein therapy in mice (33).

Finally, we observed that our immune suppressive combination therapy was also able to suppress development of AAV capsid specific antibodies. All AAV gene therapy subjects robustly develop neutralizing antibodies to the viral vector capsid, which precludes re-administration. This has implications in hepatic gene therapy for disorders requiring high expression thresholds, or in pediatric patients with dividing hepatocytes where transgene expression may be lost over time. Both rapamycin encapsulated in poly(lactic acid) nanoparticles or rapamycin + anti-CD20 combination therapy are currently being evaluated in various pre-clinical models and human clinical trials for immunomodulation to permit vector re-administration (50, 51, 59). While our findings are encouraging, it is necessary to confirm our ELISA results with the more sensitive *in vitro* cell based luciferase assay to quantitate functional anti-AAV neutralizing antibody titers.

## Data Availability Statement

The datasets generated for this study are available on request to the corresponding author.

## Ethics Statement

This animal study was reviewed and approved by University of Florida Institutional Animal Care and Use Committee, and Indiana University Institutional Animal Care and Use Committee.

## Author Contributions

MB and DM designed and coordinated all studies and wrote the manuscript. MB, BP, SK, JR, and DM conducted experiments for the study. All authors contributed to the article and approved the submitted version.

## Conflict of Interest

The authors declare that the research was conducted in the absence of any commercial or financial relationships that could be construed as a potential conflict of interest.

## Supplementary Material

Figure S1 AAV8-ApoE-hAAT-*F9* effectively eradicates inhibitors in hemophilia B mice on a BALB/c background (BALB/c-F9^−/Y^ or BALB/c-HB). BALB/c-HB mice (*n* = 8) were injected weekly with 10 IU FIX protein, week 1 IP and weeks 2–6 IV and plasma was collected on week 7 for anti-FIX IgG1 measurement by ELISA **(A)**. Four groups of BALB/c-HB mice (*n* = 8 per group) were immunized with 1 IU FIX protein in adjuvant and bled 3 weeks later to measure anti-FIX IgG1 **(B)**, Bethesda titers **(C)**, and FIX antigen levels **(D)** in plasma at indicated time points. Groups are divided into untreated controls (inverted purple triangle) and 2 × 10^9^ vg (green triangle), 1 × 10^10^ vg (red square), and 1 × 10^11^ vg (blue circle) and were followed over time as indicated. Animals received a second challenge with FIX in adjuvant at week 5 (dotted vertical line). FIX levels reported for naïve BALB/c-HB mice (orange) or inhibitor reversal mice (blue) injected with 1 × 10^11^ vg AAV8-ApoE-hAAT-*F9* vector **(D)**.

Figure S2 Anti-mCD20 treated hemophilia A mice lose FVIII memory B cells. Representative wells of a B cell ELISpot assay with unstimulated and FVIII stimulated splenocytes **(A)**. Counts of FVIII specific antibody producing cells per 1 × 10^6^ splenocytes **(B)**.
