# Supplementary figures and images for: B Cell Depletion Eliminates FVIII Memory B Cells and Enhances AAV8-coF8 Immune Tolerance Induction When Combined With Rapamycin
Source: Front Immunol. 2020 Jun 24;11:1293. doi: 10.3389/fimmu.2020.01293 (PMC7327091; doi:10.3389/fimmu.2020.01293)

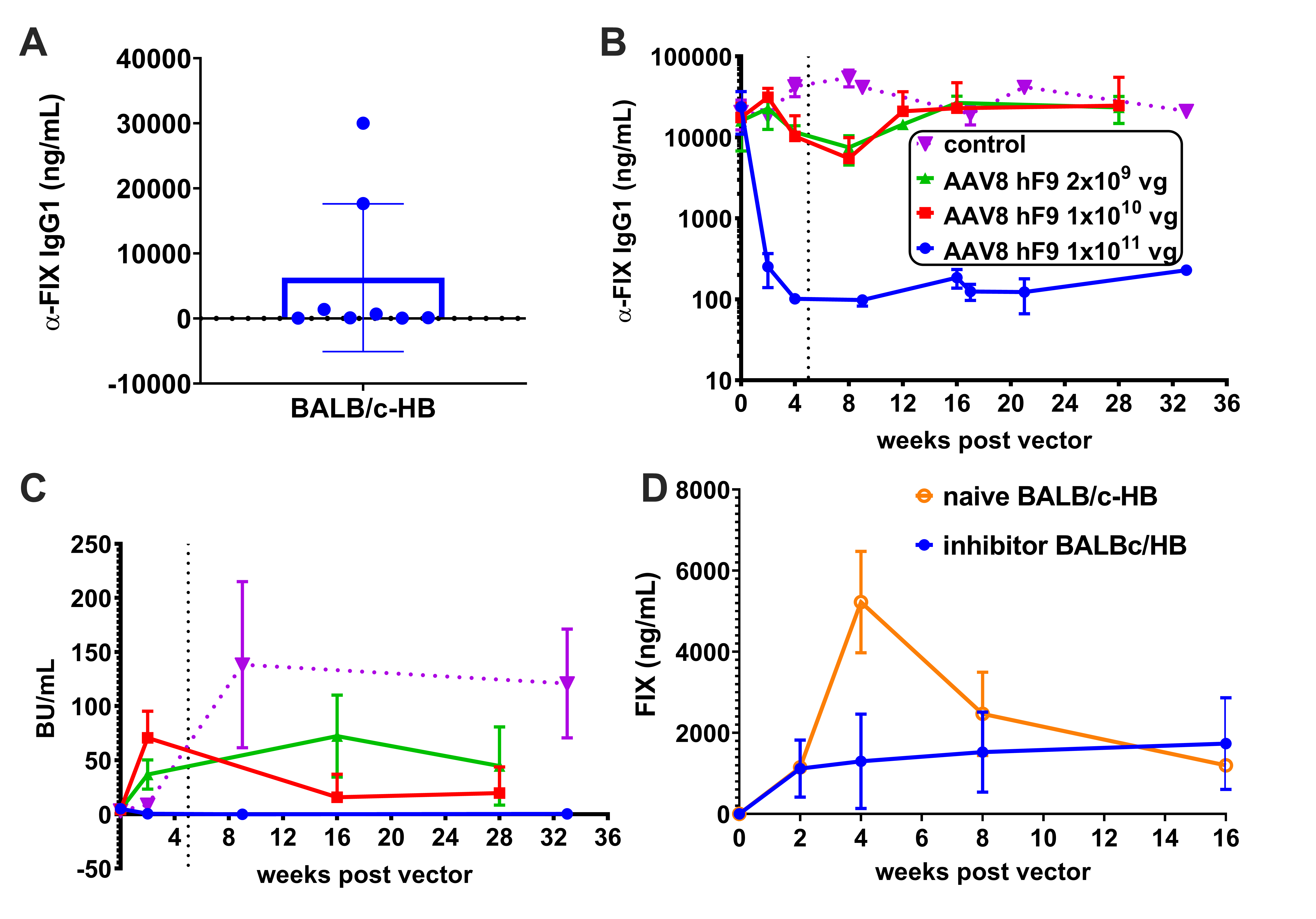

Supplement: Figure S1 — AAV8-ApoE-hAAT-F9 effectively eradicates inhibitors in hemophilia B mice on a BALB/c background (BALB/c-F9−/Y or BALB/c-HB). BALB/c-HB mice (n = 8) were injected weekly with 10 IU FIX protein, week 1 IP and weeks 2–6 IV and plasma was collected on week 7 for anti-FIX IgG1 measurement by ELISA (A). Four groups of BALB/c-HB mice (n = 8 per group) were immunized with 1 IU FIX protein in adjuvant and bled 3 weeks later to measure anti-FIX IgG1 (B), Bethesda titers (C), and FIX antigen levels (D) in plasma at indicated time points. Groups are divided into untreated controls (inverted purple triangle) and 2 × 10^9^ vg (green triangle), 1 × 10^10^ vg (red square), and 1 × 10^11^ vg (blue circle) and were followed over time as indicated. Animals received a second challenge with FIX in adjuvant at week 5 (dotted vertical line). FIX levels reported for naïve BALB/c-HB mice (orange) or inhibitor reversal mice (blue) injected with 1 × 10^11^ vg AAV8-ApoE-hAAT-F9 vector (D). [file Image_1.tif]

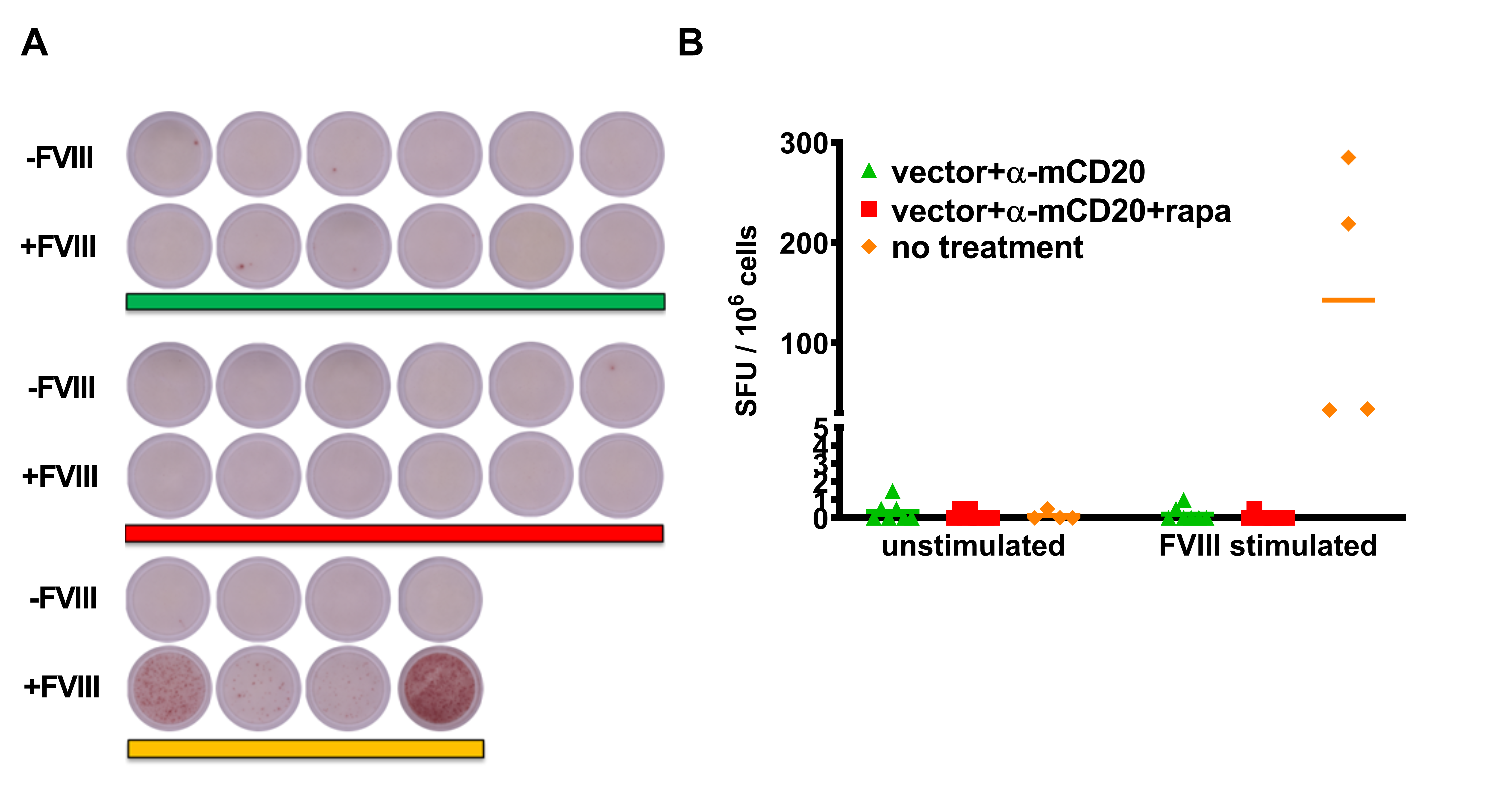

Supplement: Figure S2 — Anti-mCD20 treated hemophilia A mice lose FVIII memory B cells. Representative wells of a B cell ELISpot assay with unstimulated and FVIII stimulated splenocytes (A). Counts of FVIII specific antibody producing cells per 1 × 10^6^ splenocytes (B). [file Image_2.tif]
